# Robots in laparoscopic surgery: current and future status

**DOI:** 10.1186/s42490-019-0012-1

**Published:** 2019-05-29

**Authors:** Kenji Kawashima, Takahiro Kanno, Kotaro Tadano

**Affiliations:** 1 Tokyo Medical and Dental University, Tokyo, Japan; 2 Tokyo Institute of Technology, Tokyo, Japan

**Keywords:** Laparoscopic surgery, Surgical robot, Flexible mechanisms, Automation, Cyber-physical system

## Abstract

In this paper, we focus on robots used for laparoscopic surgery, which is one of the most active areas for research and development of surgical robots. We introduce research and development of laparoscope-holder robots, master-slave robots and hand-held robotic forceps. Then, we discuss future directions for surgical robots. For robot hardware, snake like flexible mechanisms for single-port access surgery (SPA) and NOTES (Natural Orifice Transluminal Endoscopic Surgery) and applications of soft robotics are actively used. On the software side, research such as automation of surgical procedures using machine learning is one of the hot topics.

## Background

**Fig. 1:**
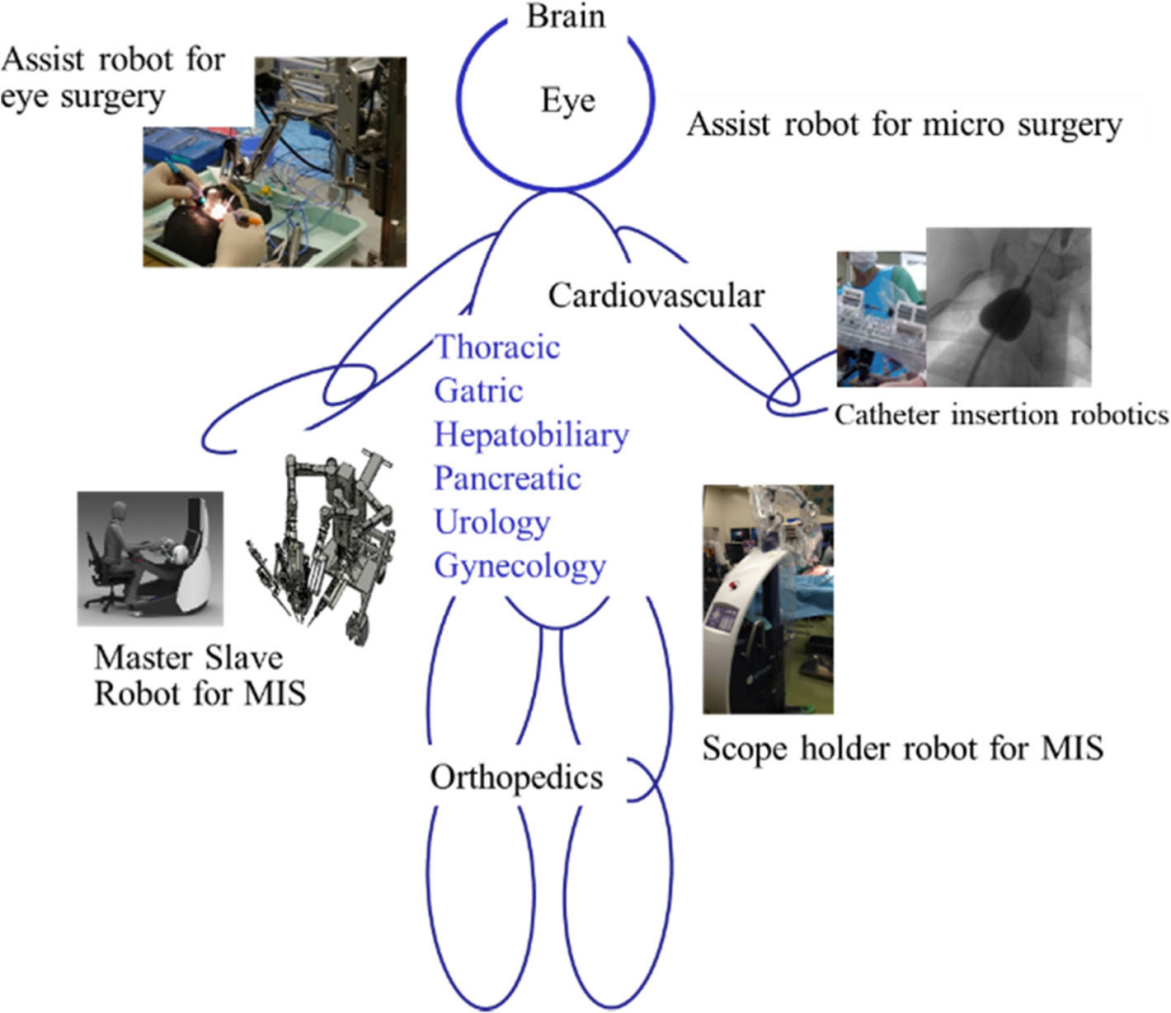
Applications of surgical robots

In recent years, surgical robots are widely used. Surgical robots are actively studied all over the world just a few decades after their introduction. The PUMA 200 robot was first used in surgery about 25 years ago, for needle placement in a CT-guided brain biopsy [1]. Research and development of surgical robots has been increasingly active since the 1990’s. In 1992, an orthopaedic surgery robot, ROBODOC, was used during a total hip replacement [2]. As a surgical robot for minimally invasive surgery (MIS), Intuitive Surgical launched the Da Vinci system in the early 2000s. Recently, surgical robots are being developed for use in many types of surgery as shown in Fig. 1 [3–6].

In this paper, we focus on robots used for laparoscopic surgery, which is one of the most active areas for research and development of surgical robots.

## Laparoscope-holder robots

Laparoscopic surgery, a group of minimally invasive surgery procedures, is improving the quality of life of patients. In the operating room, the laparoscope is maneuvered by a camera assistant according to verbal instructions from the surgeon. Laparoscopes with 3D high-definition have been commercialized. 3D vision can provide a sense of depth, which is expected while performing MIS. “Camera shake” may occur due to fatigue of the person holding the laparoscope/camera, which may cause the surgeon to lose orientation, especially when using 3D vision. Therefore, a laparoscope holder is an important and effective advancement for performing laparoscopic surgery.

**Fig. 2:**
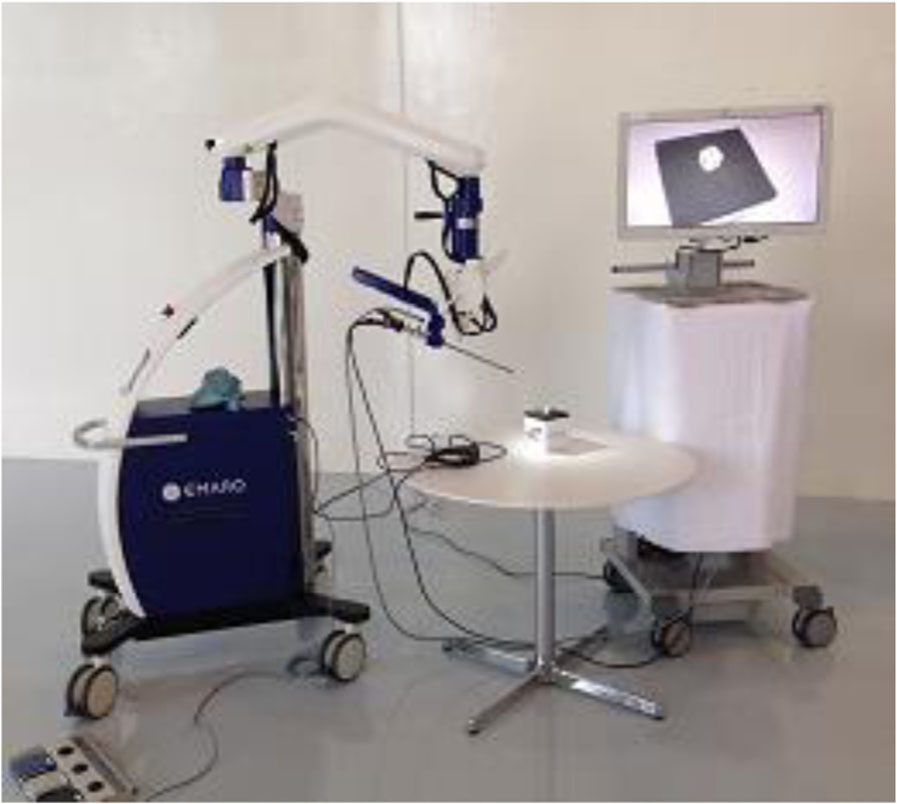
Endoscope holder robot (EMARO)

Laparoscope holders have been studied for many years, and some are commercially available. The AESOP robot was put into practical use in 1994 [7]. This is a SCARA-type robotic arm with four degrees of freedom (4 DOFs). Voice commands were added in the second version. Voice commands have the advantage that the operator’s hands remain free throughout the operation. Naviot went into clinical use in 2002 [8]. Endoscope holder robots such as FreeHand [9], Viky [10], and SOLOASSIST [11] are now commercially available. We have launched the robotic holder EMARO from a start-up venture originating in universities [12] (Fig. 2).

Previously developed robotic holders use electrical motors. However, the EMARO uses pneumatic actuators instead. Pneumatic actuators have many safety advantages such as low heat generation, compressibility, the ability to control the maximum force by regulating the supply pressure, ease of releasing the acting force by discharging the compressed air in the actuator, and the ability to develop a robotic arm that is both compact and lightweight.

**Fig. 3:**
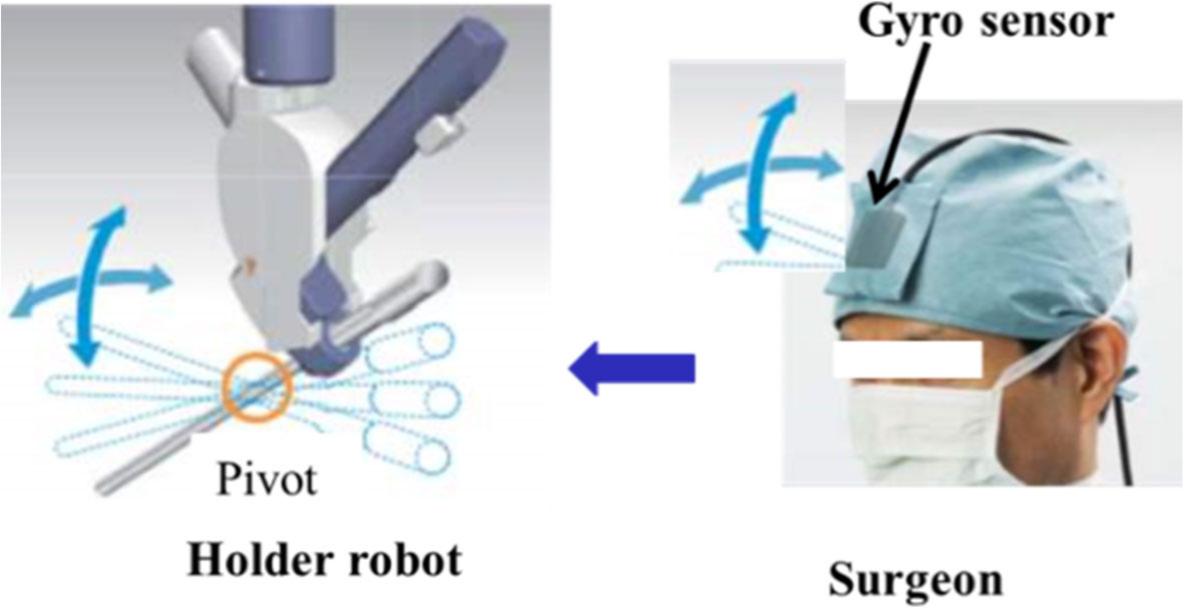
Operation of the holder robot EMARO

EMARO has 4 DOFs in total, consisting of 3 rotational DOFs around the insertion point of the trocar cannula and 1 translational DOF along the insertion direction. The movable range of pitch is from − 3° to 47°, where 0° is defined as the point where the laparoscope becomes horizontal. The movable range of yaw angle is ±90° and zoom-in and zoom-out is ±100 mm. EMARO controls the endoscope by sensing the vertical and horizontal movements of the surgeon’s head, through a gyroscope that is worn on the forehead (Fig. 3). The movement in the up/down and left/right directions are controlled by movement of the head while pushing the left foot pedal (1 of 3). The zoom in and out operations are performed by pushing the right and middle foot pedals, respectively. Five motion speeds can be selected. The effectiveness of the holder has been demonstrated in some hospitals in Japan.

## Surgical robots

Surgical robots for laparoscopic surgery can be classified into a master-slave type and hand-held forceps.

### Master-slave robots

Generally, the master-slave surgical robot has 6-degrees-of-freedom (DOF) of motion. The robot has a 4-DOF arm outside the abdominal cavity and a 2-DOF wrist joint at the tip. Therefore, the forceps tip can approach to the target in the abdomen from an arbitrary position and posture. The surgeon operates the remote slave arms with the wrist joint via the master console. The robot enables an intuitive operation since the slave arms in the abdomen reproduces the surgeon’s 6-DOF hand motion at the console. In addition, robots enable telesurgery via network and microsurgery by changing the motion scale between the master and the slave. The da Vinci surgical system is commercially successful. In 2000, the da Vinci surgery system broke new ground by becoming the first robotic surgery system approved by the FDA (US) for general laparoscopic surgery. Zeus (Computer Motion) was cleared by the FDA (US) in 2001. In 2003, Computer Motion and Intuitive Surgical merged into a single company. The latest high-end model is the da Vinci Xi. A less expensive version, the da Vinci X was also approved by the FDA. The da Vinci Sp, used for single-port surgery, has launched in the USA.

The problems in the master-slave robots are a lack of haptics (haptaesthai, from Greek for “to touch”), large size, and high cost. Open consoles, lighter instruments, and greater portability will be of continued importance for these systems. There is also a need for less invasiveness. Since the da Vinci’s basic and peripheral patents expired, research and development of surgical robots has been active.

Table 1 shows some examples of master-slave surgical robots. In USA, Google and Johnson & Johnson have invested in Verb Surgical to develop a surgical robot, although they are not shown in Table 1 because the details of this robot are not yet disclosed. In Japan, Medicaroid Co., Ltd., is the nearest to practical use. However, it is also not included in Table 1 because the details are not yet disclosed.

**Table 1:** Research and development of master-slave surgical robots

Company	Target Disease	Mechanism and Drive	Configuration	Status
Intuitive Surgical da Vinci Xi (USA)	MIS Multi-port	Link + Electrical motor	Master console and slave patient cart with four arms	FDA approved Clinical use worldwide
TransEnterix Senhanse (USA)	MIS Multi-port	Link + Electrical motor	Master console and separated slave robot arms	FDA approved
CMR surgical Verisus (UK)	MIS Multi-port	Link + Electrical motor	Master console and separated slave human like robot arms	Under development
Meere Revo-I (Korea)	MIS Multi-port	Link + Electrical motor	Master console and slave patient cart with four arms	Clinical use in Korea
RiverField (Japan)	MIS Multi-port	Flexible joint + Pneumatic	Master console and slave patient cart with arms	Under development
Intuitive Surgical da Vinci Sp (USA)	MIS Single-port	Flexible joint + Electrical motor	Master console and slave patient cart with single arm	FDA approved
Titan Medical SPORT (Canada)	MIS Single-port	Flexible joint + Electrical motor	Master console and slave patient cart with single arm	Under development
EndoMaster (Singapore)	NOTES Transoral surgery	Flexible joint + Electrical motor	Master console and slave patient cart with single arm	Clinical trial
Auris, Monarch Platform (USA)	NOTES Lung cancer	Flexible joint + Electrical motor	Master console and slave patient cart with single arm	Clinical trial

The importance of haptic feedback is widely recognized, as numbing fingers with a local anaesthetic significantly reduces grasping ability [13]. Senhanse (TransEnterix Corp.) developed a system with a force sense presentation function and has been put into practical use [14].

**Fig. 4:**
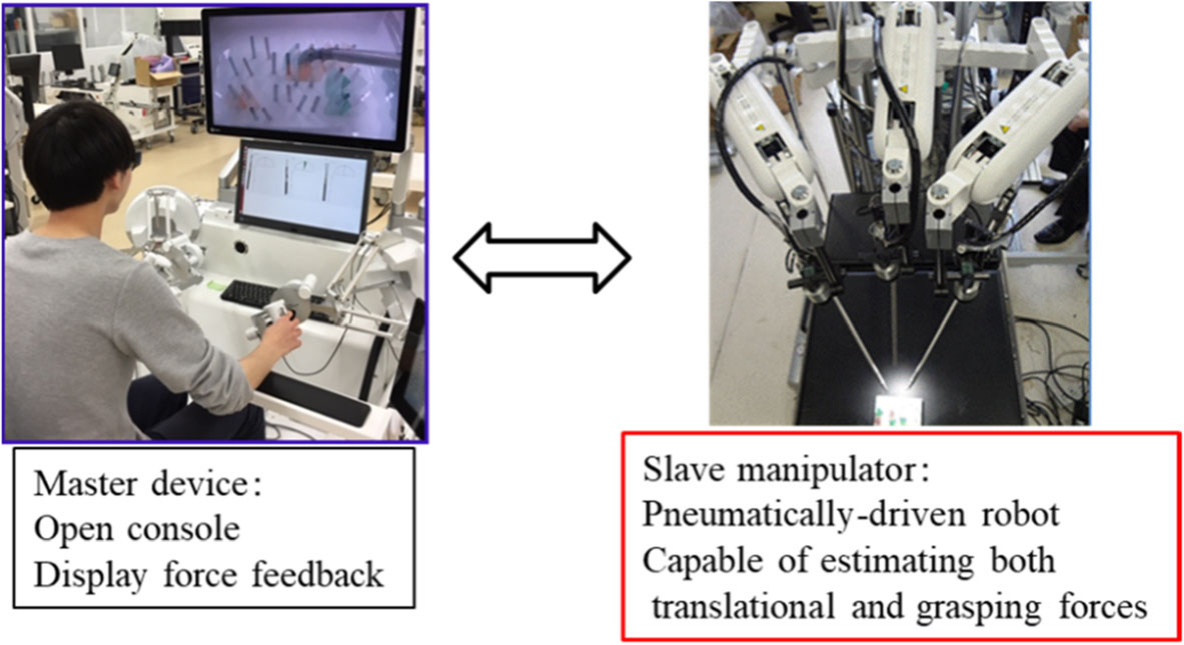
Master-slave surgical robot using pneumatic drives on the slave side

Riverfield Inc. is developing a system that uses a pneumatic drive on the slave-side, as shown in Fig. 4. The pneumatic drive makes use of the feature that the contact force and the grasping force at the forceps tip directly spring back to the pressure in the pneumatic cylinder of the drive unit. The ability to measure pressure changes with pressure sensors and estimating external force at the tip of the forceps based on this value has been implemented [15, 16]. This greatly facilitates use because the electric sensor is eliminated from the forceps tip portion which requires sterilization and cleaning. Clinical trials will be conducted in 2020.

In order to further reduce postoperative pain, risk of hernia, scarring, and formation of adhesions, surgical robots for single-port access surgery (SPA) and NOTES (Natural Orifice Transluminal Endoscopic Surgery) have been actively developed. In both types of procedures, operation of multiple instruments in a confined space is required. Therefore, as shown in Table 1, a snake-like flexible mechanism is useful for SPA and NOTES. Details can be found in ref. [6, 17].

### Hand-held robotic forceps

The master-slave robot is not the best choice for all surgical procedures since it requires space for the master console and has high introduction and operating costs [18, 19]. Hand-held robotic forceps have also been developed [20]. The forceps has a wrist joint at its tip and is manipulated from the interface mounted on the forceps. Its translation operation is the same as conventional forceps. Its setup time is shorter than the master–slave robot. The system is small because there is no master console.

The hand-held forceps can be divided into those controlled by actuators or those driven mechanically. Several electrically driven robotic forceps have been developed. Matsuhira et al. proposed robotic forceps driven by electric motors [21]. A lightweight forceps by separating actuators from the main body was developed by Focacci et al. and Hassan et al. [22]. Bensignor et al. developed a thin-diameter robotic forceps [23]. Zahraee et al. designed an interface for forceps based on ergonomics [24].

Other mechanically driven instruments have been developed [25]. Unlike the master–slave robot, hand-held robots are operated using buttons and dials, and it is difficult for surgeons to enter a complex 3-D trajectory. However, since the interface (e.g. a dial) for each axis of motion axis is independent, the surgeon is not able to operate 6-DOF and the grasper at the same time like the master–slave type. Moreover, hand-held robots are heavier than conventional forceps due to the weight of the actuators. Wearable robot forceps, mounted on the operator’s arm, is a good solution, though they have more weight for attachment parts and require a time-consuming equipment procedure [26, 27].

**Fig. 5:**
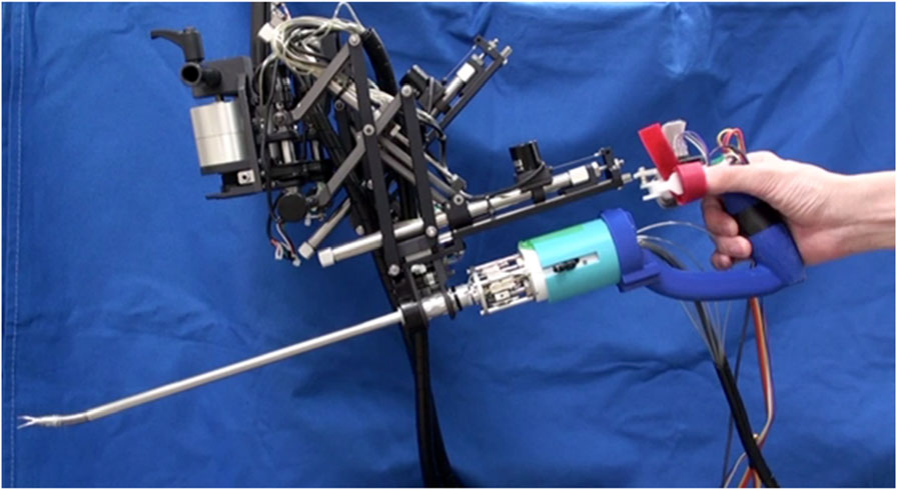
Master-slave integrated surgical robot

We have developed a robot that has operability similar to a master–slave device with the size of a handheld robot. It is a master–slave integrated surgical robot as shown in Fig. 5. The robot consists of a 2-DOF robotic forceps driven by pneumatic actuators and a 4-DOF passive holder to support the forceps. A built-in master controller enables the operation of the wrist joint of the forceps. The wrist joint and the grasper are operated like those in a master–slave robot. The translational motion is manually operated like conventional forceps. A smaller footprint is achieved by the robot than master–slave surgical robots. To reduce weight, we used pneumatic actuators that have a high power-to-weight ratio for the forceps drive. For easy insertion of a curved needle, the active motion transformation was proposed and implemented in this robot. By the precise control of the joint and an estimation of the operator’s wrist rotation, the robot enabled the transformation of rotation about the forceps sheath into rotation about the forceps tip.

## Future directions for surgical robots

Surgical robots effectively augment a surgeon’s skills to achieve accuracy and high precision during complex procedures. Use of a robot contributes to improved patient quality of life. Therefore, research and development of surgical robots will become more active.

The challenges for surgical robots include:

1. Compact and inexpensive
2. Haptic feedback to the operator
3. SPA and NOTES
4. Telesurgery
5. Applications of augmented reality
6. Automation of surgical tasks
7. Cyber-physical system coupled with robots.

We have already discussed issues 1 to 3 in the previous sections. As shown in Table 1, surgical robots with a flexible structure are providing solutions to advance the concepts of SPA and NOTES.

In ref. [28], the authors point out that telesurgery is considered a futuristic field. Stable control in teleoperation with haptic perception (Bilateral control) is being studied by many investigators [29].

It is also suggested in ref. [28] that image guidance with robotic surgery using augmented reality represents a major revolution to increase safety and deal with difficulties associated with minimally invasive approaches. Augmented reality superimposes virtual objects on the laparoscopic image or haptic feedback system, which enhances safety and efficiency of surgery [30]. For example, preoperative information such as CT image can be mixed to the real image to assist surgeons to find hidden tumor [31].

Surgeon’s fatigue can be reduced by automation and is being actively studied. In ref. [32], levels of autonomy according to the context for use are defined in six categories as “No autonomy”, “Robot assistance”, “Task autonomy”, “Conditional autonomy”, “High autonomy” and “Full autonomy”. For example, task autonomy is similar to adaptive crouise control of a vehicle, which helps some specific tasks. It involves automatic suturing and cutting. Higher-level autonomy can conduct full surgery without human operation. Except full autonomy, supervision by a human will be necessary, just like a safety driver in a car. Autonomous systems and semi-autonomous systems have started being used in surgical procedures [33, 34] and have been used for clinical applications [35].

One of the challenges in autonomous surgery is suturing task. It requires precise handling of an arc-shaped needle. Krupa et al. introduced Visual Servoing for autonomous control that brings surgical instruments to the center of the laparoscopic camera [36]. Murali et al. introduced learning by observation approach to perform autonomous tissue piercing with a needle [37]. In ref. [38], they demonstrate approaches to tie a suture autonomously using general purpose laparoscopic instruments. We proposed a system consists of a single-master and dual-slave robots [39]. The operator inserts the needle to a phantom manually using one of the slaves. Then, the other slave automatically approaches and grasps the needle.

Surgical robotics will bring surgery to the next level with the combination of robots and artificial intelligence. The existing master-slave surgical support robot is positioned as Surgery 3.0, and the next generation will be Surgery 4.0 [40]. Verb Surgical announced that Surgery 4.0 is the enabling of a digital surgical platform coupled with robots. Surgery 4.0 will help make surgery less expensive, evidence-based, easier and safer.

## Conclusion

This paper introduces developments and future directions of surgical robots for laparoscopic surgery. For robot hardware, snake like flexible mechanisms for SPA and NOTES and applications of soft robotics are actively used. On the software side, as can be seen from the concept of Surgery 4.0, research such as automation of surgical procedures using machine learning is one of the hot topics.

Various types of surgical robots will be put in practical use in the future and are expected to provide safer surgery connected with cyber space.

## Abbreviations

DOF: Degrees-of-freedom DOF
FDA: Food and Drug Administration
NOTES: Natural orifice transluminal endoscopic surgery
SCARA: Selective compliance assembly robot arm
SPA: Single-port access surgery

## References

[1] Kwoh YS (1988). A robot with improved absolute positioning accuracy for CT guided stereotactic brain surgery. IEEE Trans Biomed Eng.

[2] Pransky J (1997). ROBODOC - surgical robot success story, Indus. Robot.

[3] Davies B (2000). A review of robotics in surgery. Proc Inst Mech Eng H.

[4] Bergeles C, Yang G-Z (2014). From passive tool holders to microsurgeons: safer, smaller, smarter surgical robots. IEEE Trans Bioengineering Biomed Eng.

[5] Vitiello V (2013). Emerging robotic platforms for minimally invasive surgery. IEEE Rev Biomed Eng.

[6] Simaan N (2018). Medical technologies and challenges of robot-assisted minimally invasive intervention and diagnostics. Annu Rev Control, Robot, Auton Syst.

[7] Kraft BM (2004). The AESOP robot system in laparoscopic surgery increased risk or advantage for surgeon and patient?. Surg Endosc.

[8] Yasunaga T (2003). Remote-controlled laparoscope manipulator system, Naviot™, for endoscopic surgery. Int Congr Ser.

[9] Stolzenburg Jens-Uwe, Franz Toni, Kallidonis Panagiotis, Minh Do, Dietel Anja, Hicks James, Nicolaus Martin, Al-Aown Abdulrahman, Liatsikos Evangelos (2010). Comparison of the FreeHand® robotic camera holder with human assistants during endoscopic extraperitoneal radical prostatectomy. BJU International.

[10] Voros S (2010). ViKY robotic scope holder: initial clinical experience and preliminary results using instrument tracking. IEEE ASME Mechatronics.

[11] Kristin J (2015). Assessment of the endoscopic range of motion for head and neck surgery using the SOLOASSIST endoscope holder. Int J Med Rob Comput Assisted Surgery.

[12] Tadano K, Kawashima K (2015). A pneumatic laparoscope holder controlled by head movement. Int J Med Rob Comput Assisted Surg.

[13] Johansson RS, Westling G (1984). Roles of glabrous skin receptors and sensorimotor memory in automatic control of precision grip when lifting rougher or more slippery objects. Exp. Brain Res.

[14] Fanfani F (2016). The new robotic TELELAP ALF-X in gynecological surgery: single-center experience. Surg Endosc.

[15] Tadano K (2010). Development of a pneumatic surgical manipulator IBIS IV. J Rob Mechatronics.

[16] Miyazaki Ryoken, Kanno Takahiro, Kawashima Kenji (2019). Pneumatically driven surgical instrument capable of estimating translational force and grasping force. The International Journal of Medical Robotics and Computer Assisted Surgery.

[17] Burgner-Kahrs J (2015). Continuum robots for medical applications: a survey. IEEE Trans Robotics.

[18] Breitenstein S (2008). Robotic-assisted versus laparoscopic cholecystectomy: outcome and cost analyses of a case-matched control study. Ann Surg.

[19] Morino M (2004). Robot-assisted vs laparoscopic adrenalectomy: a prospective randomized controlled trial. Surg Endosc.

[20] Payne CJ, Yang G-Z (2014). Hand-held medical robots. Ann Biomed Eng.

[21] Matsuhira N (2003). Development of a functionalmodel for amaster-slave combined manipulator for laparoscopic surgery. Adv Robot.

[22] Focacci F (2007). Lightweight hand-held robot for laparoscopic surgery. Proc. IEEE Int. Conf. Robot. Autom.

[23] Bensignor T (2015). Evaluation of the effect of a laparoscopic robotized needle holder on ergonomics and skills. Surg Endosc.

[24] Zahraee AH (2010). Toward the development of a hand-held surgical robot for laparoscopy. IEEE/ASME Trans Mechatronics.

[25] Anderson PL, Lathrop RA, Webster RJ (2016). Robot-like dexterity without computers and motors: a review of hand-held laparoscopic instruments with wrist-like tip articulation. Expert Rev Med Devices.

[26] Awtar S (2010). FlexDex: a minimally invasive surgical tool with enhanced dexterity and intuitive control. J Med Devices.

[27] Miyazaki R, et al. Pneumatically driven handheld forceps with force display operated by motion sensor. IEEE Int Conf Robot Autom. 2015;7:604–9.

[28] Daiana M, Marescaux J (2015). Robotic Sugery. Br J Surg.

[29] Li H (2018). Operator dynamics for stability condition in haptic and teleoperation system: a survey, the international journal of medical robotics and computer assisted surgery.

[30] Pessaux P (2015). Towards cybernetic surgery: robotic and augmented reality-assisted liver segmentectomy. Langenbeck’s Arch Surg.

[31] Volonte F (2011). Augmented reality and image overlay navigation with OsiriX in laparoscopic and robotic surgery: not only a matter of fashion. H Hepatobiliary Pancreat Sci.

[32] Yang G-Z (2017). Medical robotics—regulatory, ethical, and legal considerations for increasing levels of autonomy. Science Robotics.

[33] Kranzfelder M (2013). Toward increased autonomy in the surgical or: needs, requests, and expectations. Surg Endosc.

[34] Hu D (2015). Semi-autonomous simulated brain tumor ablation with RavenII surgical robot using behavior tree. IEEE Int. Conf. On robotics and automation (ICRA).

[35] Moustris G. P., Hiridis S. C., Deliparaschos K. M., Konstantinidis K. M. (2011). Evolution of autonomous and semi-autonomous robotic surgical systems: a review of the literature. The International Journal of Medical Robotics and Computer Assisted Surgery.

[36] Krupa A (2015). Autonomous 3-D positioning of surgical instruments in robotized laparoscopic surgery using visual Servoing. IEEE International Conference on Intelligent Robots and Systems.

[37] Murali A, et al. Learning by observation for surgical subtasks: multilateral cutting of 3D viscoelastic and 2D orthotropic tissue phantoms. IEEE International Conference on Robotics and Automation. 2015:1202–9.

[38] Mayer H, et al. Human-machine skill transfer extended by a scaffolding framework. IEEE International Conference on, Robotics and Automation. International Conference paper. 2008;2866–71.

[39] Watanabe K (2018). Single master dual slave surgical robot with automated relay of suture needle. IEEE Trans Ind Electron.

[40] Feußner H, Park A (2017). Surgery 4.0: the natural culmination of the industrial revolution?. Innovation Surgical Science.

